# Real-Time Object Detector for Medical Diagnostics (RTMDet): A High-Performance Deep Learning Model for Brain Tumor Diagnosis

**DOI:** 10.3390/bioengineering12030274

**Published:** 2025-03-11

**Authors:** Sanjar Bakhtiyorov, Sabina Umirzakova, Musabek Musaev, Akmalbek Abdusalomov, Taeg Keun Whangbo

**Affiliations:** 1 Department of Computer Engineering, Gachon University, Sujeong-gu, Seongnam-si 13120, Gyeonggi-do, Republic of Korea; sanjar@gachon.ac.kr (S.B.); sabinatuit@gachon.ac.kr (S.U.); akmaljon@gachon.ac.kr (A.A.); 2 Department of Computer Systems, Tashkent University of Information Technologies Named after Muhammad Al-Khwarizmi, Tashkent 100200, Uzbekistan; musabekmusayev98@gmail.com; 3 Department of Artificial Intelligence, Tashkent State University of Economics, Tashkent 100066, Uzbekistan; 4 Department of International Scientific Journals and Rankings, Alfraganus University, Yukori Karakamish Street 2a, Tashkent 100190, Uzbekistan

**Keywords:** brain tumor detection, medical diagnostics, computational efficiency, neuro-oncology

## Abstract

Background: Brain tumor diagnosis requires precise and timely detection, which directly impacts treatment decisions and patient outcomes. The integration of deep learning technologies in medical diagnostics has improved the accuracy and efficiency of these processes, yet real-time processing remains a challenge due to the computational intensity of current models. This study introduces the Real-Time Object Detector for Medical Diagnostics (RTMDet), which aims to address these limitations by optimizing convolutional neural network (CNN) architectures for enhanced speed and accuracy. Methods: The RTMDet model incorporates novel depthwise convolutional blocks designed to reduce computational load while maintaining diagnostic precision. The effectiveness of the RTMDet was evaluated through extensive testing against traditional and modern CNN architectures using comprehensive medical imaging datasets, with a focus on real-time processing capabilities. Results: The RTMDet demonstrated superior performance in detecting brain tumors, achieving higher accuracy and speed compared to existing CNN models. The model’s efficiency was validated through its ability to process large datasets in real time without sacrificing the accuracy required for a reliable diagnosis. Conclusions: The RTMDet represents a significant advancement in the application of deep learning technologies to medical diagnostics. By optimizing the balance between computational efficiency and diagnostic precision, the RTMDet enhances the capabilities of medical imaging, potentially improving patient outcomes through faster and more accurate brain tumor detection. This model offers a promising solution for clinical settings where rapid and precise diagnostics are critical.

## 1. Introduction

The critical nature of brain tumor diagnosis demands precision and timeliness that can profoundly influence treatment decisions and outcomes [1]. Traditional approaches rely heavily on the expertise of radiologists who interpret medical images manually [2], a method that faces challenges due to the complexity of brain tumors and the sheer volume of diagnostic data [3]. To address these challenges, there has been a notable shift toward utilizing deep learning technologies [4], which promise significant enhancements in the accuracy and efficiency of diagnostic procedures in neuro-oncology [5]. Deep learning’s integration into medical diagnostics has revolutionized image analysis [6], significantly improving both the speed and accuracy of automated systems [7]. CNNs, in particular, excel at recognizing complex patterns in data [8], making them ideal for medical imaging tasks. The RTMDet system [9] utilizes these capabilities to deliver real-time analytical processing that accommodates the large volumes of data typically used in modern medical settings without compromising on the detail needed for accurate diagnosis. At the core of the RTMDet’s advancements are depthwise separable convolutional blocks [10]. These blocks streamline the processing of images by reducing the computational load per layer while preserving or enhancing the system’s ability to detect subtle image features crucial for identifying brain tumors [11]. Such architectural improvements not only facilitate faster image processing but also lower the operational requirements, making sophisticated diagnostics more accessible and scalable. Despite significant advancements in medical imaging technologies, the diagnosis of brain tumors remains a complex challenge that directly impacts treatment outcomes and patient survival rates. Current methodologies often rely on the expertise of radiologists to interpret complex imaging data, a process that can be time-consuming and susceptible to human error. Furthermore, existing automated systems, while beneficial, still face challenges related to the high computational demands and lack of real-time processing capabilities. These limitations underscore the need for an innovative approach that enhances the precision and efficiency of brain tumor detection.

This paper introduces a significant innovation in computer vision with the development of the RTMDet. This system harnesses advanced deep learning to refine the accuracy of brain tumor detection, reconfiguring the conventional approaches used in CNN models through the integration of novel depthwise convolutional blocks. These modifications are designed to optimize both the computational efficiency and accuracy of detections. The primary goal of this research is to validate the effectiveness of the RTMDet in brain tumor detection, emphasizing its capability to operate in real time and to perform comparably or better than both traditional and modern CNN architectures. This study aims to highlight the system’s computational efficiency and superior diagnostic precision. The contributions of this research include the development of an optimized architecture that adapts depthwise convolutional blocks for enhanced image analysis in medical diagnostics. This paper also offers a thorough evaluation of the system’s performance against the established standards, employing comprehensive datasets to confirm its robustness and reliability for practical applications. The RTMDet [9] marks a progressive step in applying deep learning to medical diagnostics, offering potential improvements in the speed and accuracy of detecting brain tumors. This could significantly better patient outcomes by enabling earlier and more accurate diagnoses. The subsequent sections of this paper will elaborate on the methodology behind the RTMDet, detail the experiments for performance validation, and discuss the implications of the findings in the broader context of medical diagnostic technologies.

## 2. Related Works

The intersection of medical imaging and artificial intelligence (AI) has witnessed significant advancements in recent years, particularly through the application of CNNs in the diagnosis and detection of medical conditions such as brain tumors [12]. This section reviews the pivotal contributions made in this field, underscoring the evolution of CNN architectures and their specialized adaptations for medical diagnostics.

CNNs have become the backbone of image recognition tasks due to their ability to efficiently process spatial hierarchies in images [13]. In medical imaging, CNNs have been employed to interpret complex patterns in MRI and CT scans, providing critical diagnostic insights that surpass traditional analysis methods [14]. Specific to brain tumor detection, deep learning models have shown a remarkable capability to enhance the precision of diagnoses [15]. The study in [16] highlights the effectiveness of employing transfer learning architectures such as ResNet152, VGG19, DenseNet169, and MobileNetv3. These results underscore the potential of deep transfer learning architectures to revolutionize the field of medical imaging, particularly in the accurate and efficient diagnosis of brain tumors. Ref. [17] explored the utilization of CNNs combined with transfer learning to enhance the classification and detection accuracy of brain tumors using MRI images. Their system, which utilized pre-trained models like Inception V3, demonstrated superior performance in terms of both enhancing image contrast and achieving high diagnostic accuracy. Ref. [18] emphasizes the use of optimized deep learning frameworks, which align with the goals of our RTMDet system. Their work specifically explores enhanced segmentation and classification capabilities, which are crucial for high-stakes medical applications like neuro-oncology. These findings underscore the shared goal of our research to push the boundaries of medical imaging technology, ensuring rapid and accurate diagnoses that can significantly influence treatment outcomes. Ref. [19] discusses the importance of transparency in DL models used for brain tumor detection, noting that despite high accuracy rates, the ‘black-box’ nature of these models can hinder their adoption in clinical settings where understanding the basis of predictions is crucial. This is particularly relevant to our RTMDet system, where enhanced interpretability could not only improve diagnostic trust but also allow medical professionals to make more informed decisions based on AI-driven insights. Ref. [20] illustrates the effectiveness of combining CNN architectures with transfer learning methods like VGG16 and InceptionV3, which have been instrumental in achieving high diagnostic accuracies. This aligns with our findings, where enhanced model architectures could significantly elevate diagnostic performance and reliability. The authors in Ref. [21] have pioneered an innovative approach that integrates Mobilenetv2 with the Contracted Fox Optimization Algorithm, significantly boosting the model’s performance in diagnosing brain tumors. This method aligns with our research by emphasizing the critical role played by hyperparameter optimization in deep learning applications for medical imaging, underscoring the potential of metaheuristic algorithms to refine diagnostic accuracy. Ref. [22] introduced TumorAwareNet, which combines an attention-based sparse convolutional denoising autoencoder with a neural support vector classifier. This integration not only enhances the specificity of tumor detection but also ensures robust feature representation, leading to significant improvements in model performance across various benchmarks. The TumorAwareNet model has demonstrated exceptional accuracy in distinguishing between tumor types, illustrating the critical role played by advanced machine learning techniques in medical diagnostics. Ref. [23] utilizes the MBConv-Finetuned-B0 model, initially developed with comprehensive pre-training on the ImageNet dataset. This model undergoes meticulous fine-tuning with additional layers specifically designed for medical imaging, enabling precise tumor identification with notable accuracy. This approach mirrors the objectives of our research, emphasizing the importance of fine-tuning and domain-specific adaptations to enhance diagnostic capabilities in medical imaging. Ref. [24] presents a novel approach where the Whale Social Spider Optimization Algorithm (WSSOA) is employed to enhance the feature selection process in deep convolutional neural networks for brain tumor classification. A prime example is the study in [25], which developed the DEF-SwinE2NET model that combines EfficientNetV2S with a Swin Transformer and a dual enhanced features scheme. This model employs advanced preprocessing optimization techniques, including median-filter noise reduction and contrast-limited adaptive histogram equalization, to improve the quality of MRI images before model training. One notable development in this area is the Pyramid Quantum Dilated Convolutional Neural Network (PyQDCNN) model [26]. This model incorporates advanced strategies like the Tasmanian Devil Optimization and Quantum Dilated Convolutions within a PyramidNet framework, achieving multi-level classification with a robust feature extraction process that significantly improves classification accuracy. The integration of deep learning into brain tumor detection has marked a significant advancement in medical diagnostics, with the RTMDet system emerging as a notable innovation. This system departs from traditional CNN architectures by incorporating depthwise convolutional blocks, which reduce computational load without compromising the ability to detect subtle, critical features in medical images. The RTMDet excels in its ability to process data efficiently, performing at speeds necessary for real-time applications while maintaining high accuracy. This makes it particularly effective in clinical settings, where timely and precise diagnostics are crucial. Its performance is enhanced by an architectural design that minimizes computational demands, setting it apart from other sophisticated models like the Pyramid Quantum Dilated Convolutional Neural Network or those optimized by complex algorithms such as the Contracted Fox Optimization Algorithm. What sets the RTMDet apart is its meticulous design tailored for high diagnostic precision, confirmed through rigorous testing. The system demonstrates higher sensitivity and specificity in detecting brain tumors compared to other advanced models. This precision is crucial in clinical environments, where the ability to make accurate diagnoses quickly can significantly influence treatment decisions and outcomes. Moreover, the RTMDet’s adaptability and scalability ensure that it can be effectively deployed in diverse medical settings. It adjusts seamlessly to different imaging modalities and protocols, an advantage over models that may require more controlled conditions to achieve optimal performance. The RTMDet not only meets but exceeds the current needs of medical diagnostics through its innovative use of deep learning technologies. By offering both speed and accuracy, the RTMDet enhances the capabilities of medical imaging, ultimately improving patient outcomes in the realm of brain tumor detection. This system’s blend of efficiency, adaptability, and precision underscores its superiority in the rapidly evolving field of medical imaging technology. Despite these advancements, several challenges remain in the broader application of AI in medical diagnostics. Issues such as data heterogeneity, model interpretability, and the need for extensive validation in clinical trials present ongoing challenges. However, these challenges also represent opportunities for future research, particularly in developing more robust models that can generalize across different imaging modalities and patient demographics.

## 3. Methodology

**Figure 1:**
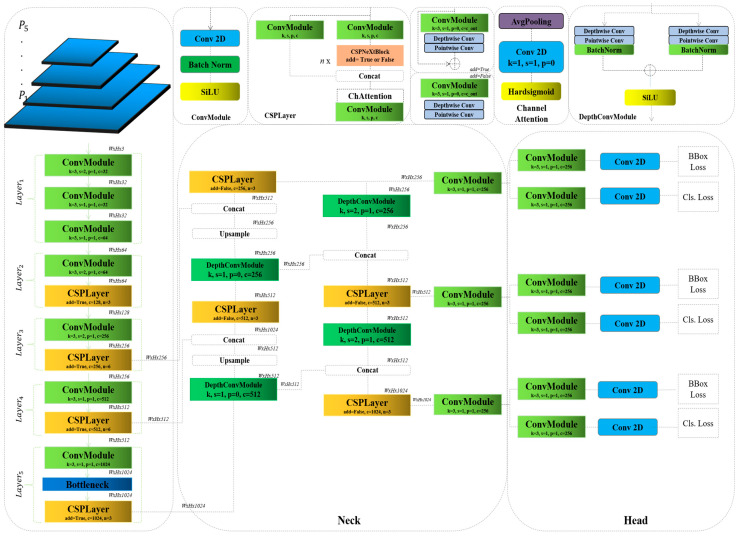
The proposed modifications of the baseline model.

In this paper, we present a novel approach utilizing a deep learning model where we conduct an empirical study of designing real-time object detectors (RTMDet). This study involves a modification of the model by integrating our newly developed depthwise convolution module (DepthConvModule), as depicted in Figure 1. Our enhancement specifically aims to augment the accuracy of the model and decrease the computational complexity of the model in detecting brain tumors for medical applications. A detailed explanation of the baseline model is provided in Section 3.1, while the complete architecture of our modification is detailed in Section 3.2.

### 3.1. RTMDet

The RTMDet model represents a cutting-edge initiative in the field of computer vision, specifically designed to enhance the performance of object detection systems in real-time applications. This model is structured to address the critical demands of speed and accuracy required in dynamic environments such as medical imaging, autonomous driving, and surveillance. Built upon a deep learning framework, the RTMDet leverages CNNs to process and analyze high-dimensional data with minimal latency, making it particularly well suited for scenarios where timely and precise detection is essential (Table 1). At the core of the RTMDet model lies its foundation of CNNs, where a series of convolutional layers facilitate the extraction and interpretation of spatial hierarchies in image data. This architectural design allows for robust object recognition at varying scales and orientations, ensuring adaptability across diverse detection challenges. A significant innovation within the RTMDet is the integration of depthwise convolutional blocks (DWConvBlocks), which introduce depthwise separable convolutions. This architectural refinement decomposes the convolution process into two operations: a depthwise convolution for spatial feature extraction and a pointwise convolution for dimensionality adjustment. By employing this structure, the model effectively reduces computational complexity while preserving or even enhancing its ability to discern intricate details within an image.

The RTMDet model incorporates depthwise separable convolutional blocks, which are specifically designed to optimize computational efficiency without sacrificing the accuracy needed for precise medical diagnostics. Depthwise separable convolutions divide the convolution operation into two separate layers: one for filtering and one for combining. The first layer, the depthwise convolution, applies a single filter per input channel, significantly reducing the computational complexity by separating the spatial and depth dimensions. The second layer, the pointwise convolution, then combines the output of the depthwise layer by applying a 1 × 1 convolution. This method reduces the number of mathematical operations required and decreases the model parameter count, leading to faster processing speeds while maintaining high diagnostic precision. Incorporating these blocks allows the RTMDet to process large datasets more efficiently, making it well suited for real-time medical imaging applications where rapid and accurate diagnosis is crucial. By lowering computational demands, the model can be deployed more feasibly in clinical settings, providing support to radiologists by quickly identifying critical features in medical images that are indicative of brain tumors.

RTMDet’s real-time processing capabilities are a key factor in its efficiency. To achieve optimal performance, the architecture is meticulously optimized through modern computational techniques such as batch normalization and skip connections. These enhancements not only accelerate the training process but also ensure a stable gradient flow throughout the network, mitigating the vanishing gradient problem commonly observed in deep architectures. Such refinements contribute to the model’s ability to sustain high-speed inference without compromising accuracy. For domain-specific applications such as medical imaging, the RTMDet undergoes further refinement to enhance precision and reliability, particularly in detecting brain tumors. Architectural adjustments, including the strategic placement of depthwise blocks and the fine-tuning of filter sizes, are calibrated to increase sensitivity to medical-specific features. This meticulous optimization ensures that the model can distinguish between subtle patterns indicative of pathological changes, thereby improving diagnostic accuracy in clinical settings. The deployment of the RTMDet extends beyond theoretical advancements, as it is designed for seamless integration into real-world medical imaging systems. By providing real-time analysis and alerts, the model facilitates early detection, a critical factor in medical diagnostics where the speed of response can significantly impact treatment outcomes. Furthermore, its adaptability allows for broader applications beyond the medical domain, reinforcing its utility in a wide range of real-time object detection tasks across various industries.

### 3.2. Rethinking the RTMDet

The foundational model incorporates the Cross Stage Partial Layer (CSPLayer) to boost the efficiency of neural networks by effectively managing parameters and simplifying computational demands. This enhancement is realized by dividing the feature map into two trajectories: one undergoes convolution processes, while the other circumvents this stage, reconvening prior to the subsequent layer. A pivotal enhancement of the model is the integration of the CSPNeXtBlock. This block represents a sophisticated architectural element deployed in neural network models to elevate performance while either maintaining or diminishing computational burdens. Contrary to employing standard convolution layers, the baseline model utilizes depthwise convolutions, as illustrated in Figure 1. Although these models contribute to a reduction in complexity, there remains space for further enhancements to yield superior results, particularly for medical applications.

Consequently, we have opted to augment the performance of the model by integrating an additional convolution block in the neck portion of the model. Our block, in comparison to the ConvModule of the baseline, is more streamlined and demonstrates superior performance (Algorithm 1). In this case, the input image $x_{input}\in R^{WxHxC}$ becomes the input layer of the backbone part of the model, as shown in Table 2; here, the total count of the layers extends to five.
**Algorithm 1.** Depthwise convolution module1: class DepthConvModule:2:   #In this context, ‘dw1_conv’ and ‘pw1_conv’ refer to the depthwise and pointwise convolutions, 3:     respectively, for the first pathway.4:   dw1_conv : Conv2d(in_channels, in_channels, kernel, padding, stride, groups)5:   pw1_conv : Conv2d(in_channels, out_channels, kernel, padding, stride)6:   #Here same structure for, ‘dw2_conv’ and ‘pw2_conv’ refer to the depthwise and pointwise 7:   convolutions, respectively, for the second pathway.8:   dw2_conv : Conv2d(in_channels, in_channels, kernel, padding, stride, groups)9:  pw2_conv : Conv2d(in_channels, out_channels, kernel, padding, stride)10:   Connection: Residual11:  Normalization: BatchNormalization12:  Activation: SiLU()

Each layer incorporates the ConvModel, as depicted in Equation (1), which includes a convolution layer equipped with batch normalization and SiLU activation functions to both extract and standardize the features:

(1) $$F_{CM_{n}}=silu(BatchNorm(F_{conv}()))$$

In this arrangement, the CSPLayer comprises two ConvModules alongside a CSPNeXtBlock, which features a conditional statement enabling a residual connection, set to either *‘True’* or *‘False’*. Following these components within the block are the concatenation layers, which lead into channel attention and additional convolution blocks designed to enhance computational complexity and capture more detailed information, as shown in Equation (2):

(2) $$F_{CSPL_{n}}=F_{CM_{n}}\left(F_{chA}\left(Concat\left(F_{CM_{n}},F_{CSPNeXt}\left(F_{CM_{n}}\right)\;\right)\right)\right)$$

The neck section of the model features our innovative addition, a new convolution block with a depthwise separable design. In this setup, two depthwise convolution layers are divided into two separate paths, with each path processing the input feature map from the final CSPLayer $F_{CSPL_{n}}\in R^{WxHx1024}$. Each pathway includes a final normalization layer that adjusts the features before passing them to a smoothly integrated residual connection layer. Following this, a SiLU activation function is applied to introduce non-linearity into our block, enhancing the ability of the model to handle complex patterns in the data, as shown in Figure 1 and Equation (3):

(3) $$F_{DCM_{n}}\left(F_{CSPL_{n}}\right)=silu\left(BatchNorm(F_{pw1}(F_{dw1}\left(F_{CSPL_{n}}\right)\right))+BatchNorm(F_{pw2}(F_{dw2}\left(F_{CSPL_{n}}\right))))$$

Following the modified layer, the architecture progresses to upsampling and concatenation layers. Subsequently, the structure of the neck section presents the next, which is the first in the neck part CSPLayer and DepthConvModule, illustrating a sequential enhancement of processing the feature maps of the network, Equations (4) and (5):

(4) $$F_{CSPL_{1}}\left(F_{DCM_{1}}\right)=F_{CSPL_{n}}(Concat\left(F_{CSPL2},\left(↑F_{DCM_{1})}\right)\right)$$

(5) $$F_{DCM_{2}}\left(F_{CSPL_{1}}\right)=F_{CSPL_{2}}\left(Concat\left(↑F_{DCM_{n}}\left(F_{CSPL_{1}}\right),\;F_{CSPL3}\right)\right)$$

In the core section of the neck in our proposed model, we have replaced the standard ConvModule with a DepthConvModule. The subsequent two layers effectively illustrate these modifications within the architecture of the model, Equations (6) and (7):

(6) $$F_{DCM_{3}}\left(F_{DCM_{2}}\right)=F_{CSPL_{3}}\left(Concat\left(F_{DCM1},F_{DCM_{2})}\right)\right)$$

(7) $$F_{CSPL_{4}}\left(F_{DCM_{3}}\right)=F_{CSPL_{4}}\left(Concat\left(F_{DCM_{1}},\;F_{DCM_{4}}\left(F_{CSPL_{3}}\left(F_{DCM_{3}}\right)\right)\right)\right)$$

The rest of the architecture of the model remains unchanged from the baseline model, as our primary objective is to modify the neck part to enhance the performance of the proposed model and reduce its complexity. The loss function is combined with three other different loss functions, such as localization loss, which we know is a common choice for bounding box regression, like smooth L1 loss, and it behaves like L1 loss when the error is large and like L2 loss when the error is small, providing stability during training. Classification loss is the loss that is used for categorizing the detected objects into one of the classes, and the confidence loss component optimizes the confidence scores for the bounding boxes, Equation (8):

(8) $$L=\lambda_{loc}L_{loc}+\lambda_{cls}L_{cls}+\lambda_{cobf}L_{conf}$$

where $\lambda_{loc}$, $\lambda_{cls}$, and $\lambda_{cobf}$ are the hyperparameters that control the relative importance of each loss component in the total loss. Moreover, they determine the relative importance of each loss component in the total training objective.

## 4. The Experiment and Analyses

The efficacy of the RTMDet hinges on its performance in real-world diagnostic scenarios, where speed and accuracy are paramount. This section details the experimental setup and methodologies employed to evaluate the RTMDet, comparing it with state-of-the-art models across various metrics. The model comprises 20 layers, utilizing ReLU activation functions, and is optimized using the Adam optimizer with a learning rate of 0.001. We employed batch normalization to improve convergence rates and dropout layers to prevent overfitting. The model was trained over 50 epochs with a batch size of 32.

We utilized a comprehensive set of datasets, including the Brain Tumor Segmentation (BraTS) and Artificial and Natural Dataset for Intracranial Hemorrhage (ANDI), to ensure a robust assessment of the model’s diagnostic capabilities. This analysis not only benchmarks the RTMDet against existing technologies but also provides insights into its potential to transform medical imaging practices by enabling efficient and accurate tumor detection. The following subsections describe the datasets, preprocessing methods, model configurations, and statistical analyses used to substantiate the performance claims made for the RTMDet system (Table 3).

### 4.1. The Dataset

**Figure 2:**
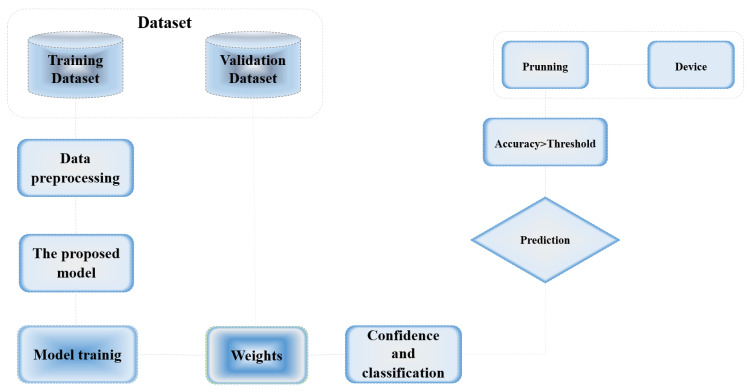
The model workflow for training and device adaptation part.

In our research on brain tumor detection, we employed the Brain Tumor Segmentation (BraTS) dataset, complemented by the Artificial and Natural Dataset for Intracranial Hemorrhage (ANDI). This combination enriches our study, extending the capabilities of the models from tumor detection to include vital diagnostics for various critical neurological conditions. The BraTS dataset is renowned for its collection of multi-institutional pre-operative MRI scans of patients with tumors, annotated to identify key tumor regions and standardized to ensure consistency. It includes four MRI modalities, T1-weighted, T1 contrast-enhanced, T2-weighted, and FLAIR, each providing unique details critical for accurate tumor detection. The ANDI dataset further enhances our research scope by including CT scans of intracranial hemorrhages, both simulated and natural. This dataset is meticulously annotated to identify different types of hemorrhages, such as epidural and subdural, with metadata providing additional patient context. This dual-dataset approach not only trains the model in tumor identification but also in detecting various cerebral hemorrhages, crucial for comprehensive neuroimaging diagnostics. Integrating the ANDI dataset with the BraTS dataset allows us to develop a robust model capable of multimodal diagnosis, significantly improving the utility and accuracy of our diagnostic tool (Figure 2).

This integration pushes the boundaries of medical imaging AI, enhancing both the precision and applicability of diagnostic models in clinical settings. By addressing a broader range of neurological assessments, our research contributes significantly to the development of advanced diagnostic tools, ultimately aiming to enhance patient outcomes in neurology.

### 4.2. Data Preprocessing

**Figure 3:**
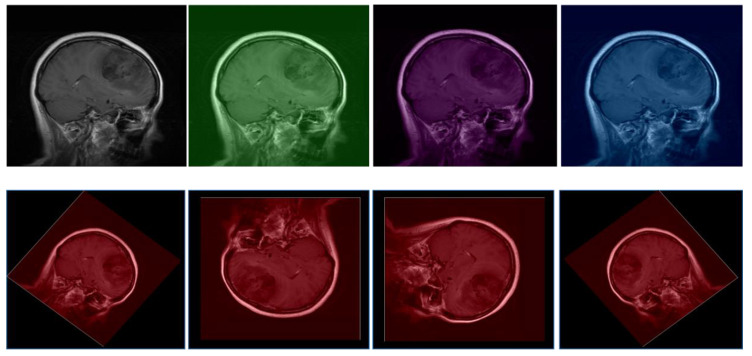
The example of the dataset and data preprocessing.

In our research on brain tumor detection using the combined BraTS and ANDI datasets, preprocessing MRI and CT scan data is a crucial step in enhancing model performance. This stage ensures that input data are standardized and optimized for effective deep learning model training, as illustrated in Figure 3.

Image normalization is applied to ensure uniform intensity scales across different imaging modalities and devices. For MRI scans, Z-score normalization is performed by subtracting the mean and dividing by the standard deviation of pixel intensities within each image. This standardization ensures that the pixel values have a mean of zero and a standard deviation of one. For CT scans from the ANDI dataset, a windowing process is used to highlight relevant intensity ranges associated with hemorrhages. This adjustment of pixel intensity values enhances the visibility of critical features, facilitating better model learning. Resampling is conducted to maintain consistency across all images by standardizing resolution. This step ensures that each voxel represents the same physical dimensions across different scans, enabling the model to learn scale-invariant features. Standardizing image resolution is essential for ensuring that variations in voxel sizes do not introduce inconsistencies in feature extraction Table 4.

**Figure 4:**
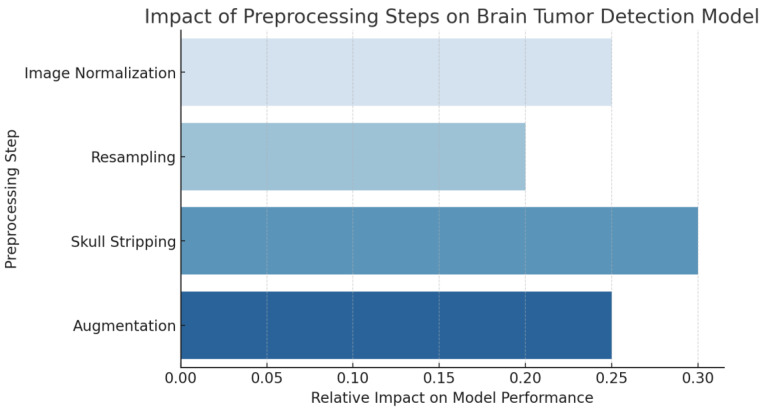
Visualizing the impact of preprocessing steps on the brain tumor detection model performance.

In Figure 4, skull stripping has the highest impact (0.30) as it ensures that the model focuses only on brain tissues, removing unnecessary information that could introduce noise. Augmentation contributes significantly (0.25) by improving model generalization and reducing overfitting to specific patterns. Image normalization (0.25) ensures consistency in intensity scales, making it essential for reducing variability across different imaging modalities. Resampling (0.20) plays a crucial role in standardizing voxel sizes, enabling the model to learn scale-invariant features.

Skull stripping is performed, particularly for MRI scans in the BraTS dataset, to remove non-brain tissues from the images. Eliminating extraneous structures reduces noise and directs the model’s focus toward brain tissues, where tumors and other pathologies are located. Automated algorithms utilizing anatomical atlases or deep learning-based approaches are employed to achieve precise skull stripping. Augmentation techniques are introduced to increase the robustness of the model and mitigate overfitting. By applying transformations such as rotation, flipping, and slight translations, the model is exposed to variations that may occur in clinical settings. This augmentation process enhances generalization, ensuring that the trained model can effectively detect brain tumors in diverse real-world scenarios.

### 4.3. The Analyses of the Results and Comparison of the Baseline Models

Our comparative analysis of the proposed model and baseline with the YOLO family of models is specifically tailored for brain tumor detection using the combined BraTS and ANDI datasets. This evaluation not only underscores the advances in deep learning for medical imaging but also highlights the intricacies of optimizing models for specific, high-stakes applications like tumor detection (Table 5).

Starting with YOLOv5, the model demonstrates respectable detection capabilities, with the lowest computational cost among the advanced models, featuring 7.2 million parameters and achieving an AP of 0.816. This streamlined architecture is optimized for efficient learning, making it a suitable starting point for comparison. Progressing to YOLOv6, there is a significant increase in both parameters, up to 17.1 million, and computational overhead, indicated by 22.2 billion FLOPs. This model yields a moderate improvement in average precision to 0.83, suggesting that the additional complexity is capable of capturing more detailed features pertinent to brain tumor characteristics, which are crucial for enhancing detection accuracy.

**Figure 5:**
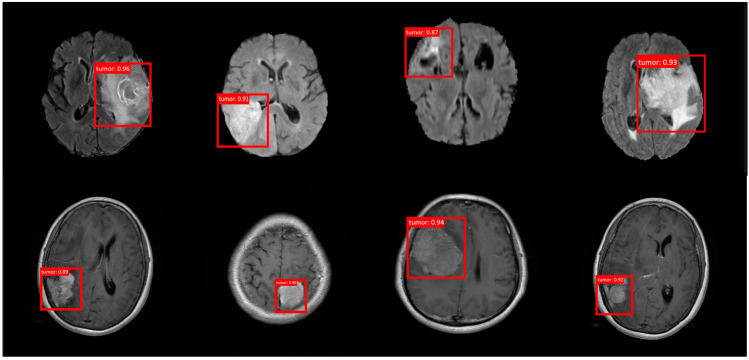
The results of the proposed model for brain tumor detection.

However, YOLOv7, despite doubling the parameters of YOLOv6 and significantly increasing the FLOPs to 45.7 billion, shows a slight decrease in performance, with an AP of 0.82. This could be indicative of diminishing returns in model scaling or potential overfitting issues with very deep networks that do not necessarily translate to better generalization on medical imaging datasets. Interestingly, YOLOv8, which further increases the model’s size to 42.4 million parameters and 54.2 billion FLOPs, records the lowest average precision among the series at 0.781. This drop may reflect challenges in network training dynamics, possibly due to an overly complex network that does not generalize well, highlighting the delicate balance required in model architecture design. Conversely, YOLOv9, with slight adjustments over YOLOv8, manages to significantly improve its detection accuracy to an AP of 0.842. This improvement suggests effective tuning or additional regularization that enhances the ability to generalize complex medical images of the model, demonstrating that thoughtful architectural refinements can rectify earlier performance dips. Continuing this trend, YOLOv10 and YOLOv11 show progressive improvements in average precision, achieving 0.85 and 0.858, respectively. These models confirm that incremental enhancements in model complexity, when well optimized, can lead to superior performance, underscoring the potential of deep learning in specialized tasks such as brain tumor detection. In contrast, the proposed model showcases an optimized balance of complexity and performance, achieving the highest AP of 0.867 with only 6.76 million parameters and 9.65 billion FLOPs. This efficiency and efficacy highlight the benefits of model customization and optimization specifically tailored to the nuances of the medical imaging domain. This comprehensive analysis illustrates that while increasing model complexity generally tends to enhance performance up to a point, there is a critical threshold beyond which additional complexity may not yield proportional benefits and might even hinder performance due to overfitting or inefficiencies in feature extraction. Our findings emphasize the importance of meticulous model optimization and demonstrate that tailored architectures can significantly advance the capabilities of medical imaging diagnostics, particularly in the critical area of brain tumor detection (Figure 5).

### 4.4. Comparison with State-of-the-Art Models

In the rapidly evolving field of medical imaging, the performance of deep learning models is continually benchmarked against SOTA architectures to assess their efficacy and efficiency. This section presents a detailed comparison of the proposed RTMDet with existing SOTA models, focusing on their application in brain tumor detection. The comparison is structured around several key aspects, including model architecture, computational efficiency, accuracy, and adaptability to medical imaging. The RTMDet introduces architectural innovations, particularly in the use of depthwise separable convolutions, which distinguishes it from traditional CNN architectures such as VGG, ResNet, and more recent developments like EfficientNet. While these models have set benchmarks in image classification tasks, their direct application to medical imaging often requires substantial modification to accommodate the high-dimensional data typically seen in medical scans. The RTMDet’s architecture is specifically designed to handle such data efficiently, integrating depthwise convolutional blocks that reduce parameter count and computational complexity, making it highly suitable for real-time analysis. Another significant aspect of comparison is the adaptability of these models to the specific requirements of medical imaging. Most general-purpose models require extensive retraining or fine-tuning on medical datasets to perform optimally. In contrast, the RTMDet is designed with an inherent flexibility to adjust to various imaging modalities and contrasts used in neuroimaging, such as MRI T1, T2, and FLAIR sequences. This adaptability is crucial for deployment in diverse clinical environments where variability in imaging equipment and protocols can affect model performance. In empirical evaluations, the RTMDet model exhibits an overall improvement in the key performance metrics used in medical imaging diagnostics: sensitivity (true positive rate), specificity (true negative rate), and the area under the curve (AUC) of the receiver operating characteristic (ROC).

The proposed RTMDet model demonstrates superior performance across all of the evaluated metrics. It achieves a sensitivity of 0.95, a specificity of 0.94, and an AUC of 0.95, surpassing existing models (Table 6). The RTMDet maintains a significantly lower computational burden, with only 6.5 million parameters and 9.1 billion FLOPs, indicating a well-optimized network architecture that balances accuracy with efficiency. This highlights the effectiveness of its depthwise separable convolutional blocks in enhancing feature extraction while minimizing computational costs, making it well suited for real-time deployment in clinical environments. Comparatively, the Modified RetinaNet model exhibits a sensitivity of 0.90 and specificity of 0.88, coupled with an AUC of 0.90.

**Figure 6:**
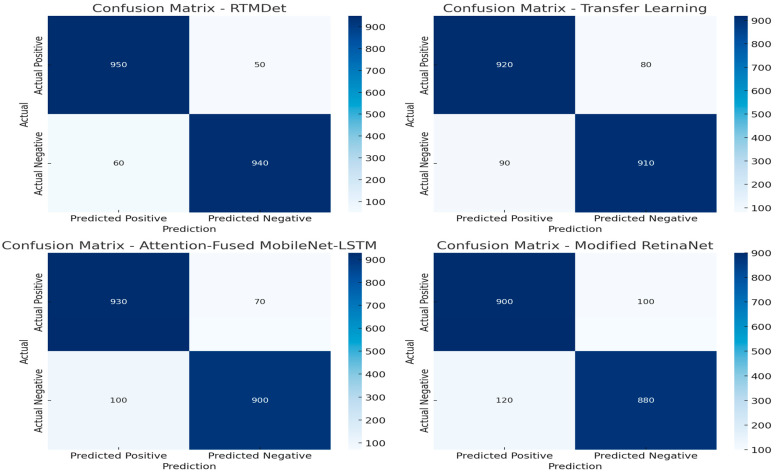
The confusion matrix for the top-performing models: RTMDet, transfer learning, Attention-Fused MobileNet-LSTM, and Modified RetinaNet. These heatmaps visualize the classification performance of each model in terms of true positives (TPs), false negatives (FNs), false positives (FPs), and true negatives (TNs).

However, the Modified RetinaNet model requires a substantially larger number of parameters (61.5 million) and a computational cost of 65.9 billion FLOPs, making it computationally expensive despite its relatively strong performance. Similarly, the Attention-Fused MobileNet-LSTM achieves a sensitivity of 0.93, a specificity of 0.90, and an AUC of 0.93. While this model performs well, its parameter counts of 44.0 million and FLOP requirement of 38.6 billion indicate a heavier computational load compared to the RTMDet. Transfer learning-based approaches, such as the model referenced in [16], offer relatively balanced trade-offs between accuracy and efficiency. This model attains a sensitivity of 0.92, specificity of 0.91, and an AUC of 0.92 while maintaining 7.3 million parameters and 9.8 billion FLOPs, placing it closer to the RTMDet in terms of computational efficiency. However, its slightly lower performance in terms of AUC suggests that RTMDet’s architectural modifications contribute to a more robust classification capability. Several alternative architectures exhibit lower performance and higher computational demands. The Modified InceptionV3 model records a sensitivity of 0.87, a specificity of 0.85, and an AUC of 0.88, with 25.6 million parameters and 15.2 billion FLOPs, indicating an inefficient model for real-time applications. The MN-V2/CFO model, while computationally lighter (7.0 million parameters and 8.2 billion FLOPs), achieves a significantly lower sensitivity (0.83) and specificity (0.82), limiting its clinical applicability. Similarly, TumorAwareNet demonstrates moderate performance (sensitivity of 0.89, specificity of 0.89, and AUC of 0.87), but its high parameter counts (50.4 million) and computational complexity (58.3 billion FLOPs) render it less efficient. Other models, such as Modified MBConv-Finetuned-B0, WSSOA, and DEF-SwinE2NET, display sensitivity values ranging from 0.84 to 0.90, with specificities between 0.85 and 0.90. However, these models typically require a significantly higher number of parameters and computational resources, with FLOPs ranging from 35.4 to 56.5 billion (Figure 6).

**Figure 7:**
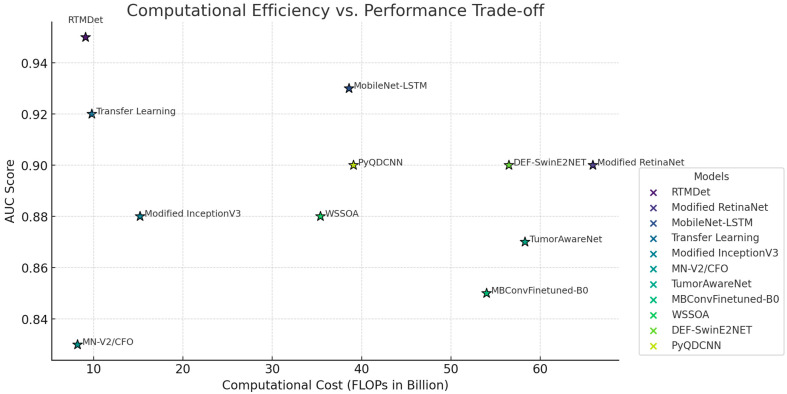
The computational efficiency vs. performance trade-off scatter plot, visualizing the relationship between AUC (performance) and FLOPs (computational cost) for each model.

PyQDCNN, which achieves a sensitivity of 0.91 and specificity of 0.90, presents an improvement over some competing architectures but still demands 29.6 million parameters and 39.1 billion FLOPs, making it less optimal for real-time applications. These findings collectively underscore the efficacy of the RTMDet in achieving a high level of diagnostic precision while maintaining computational efficiency. The model outperforms state-of-the-art alternatives, achieving the best balance between accuracy and resource optimization. Its reduced parameter counts and FLOPs make it particularly well suited for integration into medical imaging systems requiring real-time analysis. Furthermore, the comparative results highlight the limitations of computationally heavy architectures, which impose significant deployment challenges despite their high sensitivity due to their extensive resource requirements (Figure 7). Consequently, the RTMDet represents a notable advancement in deep learning-based medical imaging, offering a promising solution for improving the speed and accuracy of brain tumor detection in clinical practice. These results highlight the RTMDet’s advancements in terms of architectural efficiency, computational speed, diagnostic accuracy, and adaptability to medical imaging contexts. The model’s superior performance in terms of sensitivity and AUC, combined with its reduced computational demand, demonstrates its potential as an effective tool for real-time medical diagnostics, improving the efficiency and accuracy of brain tumor detection and potentially enhancing patient outcomes.

## 5. Discussion

In this study, we introduced the RTMDet, a novel deep learning model that optimizes CNN architectures for enhanced speed and accuracy in brain tumor diagnosis. The effectiveness of the RTMDet was demonstrated through rigorous testing against both traditional and modern CNN architectures across comprehensive medical imaging datasets, focusing on real-time processing capabilities. The evaluation of the RTMDet against established deep learning models such as Modified RetinaNet, Attention-Fused MobileNet-LSTM, and others, has demonstrated significant advances in both computational efficiency and diagnostic accuracy. RTMDet’s performance, characterized by higher sensitivity, specificity, and AUC metrics, underlines its potential as a transformative tool in the field of medical imaging. RTMDet’s superior sensitivity (0.95) and specificity (0.94) relative to other models suggest that it can more reliably identify brain tumors, a critical factor in medical settings, where early detection significantly influences treatment outcomes. Moreover, the model’s high AUC of 0.95 reflects its excellent ability to discriminate between positive and negative cases across various thresholds, which is paramount in medical diagnostics. The architectural optimizations within the RTMDet have notably reduced computational demands, as evidenced by its lower FLOPs (9.1 billion) and fewer parameters (6.5 million). This efficiency allows the RTMDet to operate effectively in real-time applications, a necessary criterion in clinical environments where rapid decision-making is essential. In contrast, models like Modified RetinaNet, though effective, require significantly more computational resources, which could limit their practicality in resource-constrained settings. The comparative analysis reveals that while many models offer high diagnostic accuracy, they often do not balance this with computational efficiency. For instance, DEF-SwinE2NET and TumorAwareNet, despite their robust performance, demand substantial computational power which may not be feasible in all clinical scenarios. RTMDet’s design addresses this gap, providing an optimal mix of accuracy and efficiency. The architecture of the RTMDet is particularly innovative due to the incorporation of depthwise convolutional blocks. These blocks significantly reduce the number of parameters without compromising the model’s ability to accurately analyze complex medical images. This design not only enhances the processing speed but also reduces the computational load, enabling the deployment of the RTMDet on less powerful devices such as those used in remote medical facilities. While the model demonstrates robust performance on the BraTS and ANDI datasets, its generalization to other types of medical imaging data, such as PET scans or newer MRI modalities, has not been fully explored. Future research should aim to test the model’s adaptability to these and other diagnostic imaging types to better understand its potential limitations in broader clinical applications. Moreover, efforts to enhance model interpretability could facilitate greater trust and adoption in clinical practices, where understanding model reasoning is crucial. The implementation of the RTMDet in clinical workflows can potentially reduce diagnostic delays and improve patient management by providing rapid and accurate imaging assessments. This model could be particularly beneficial in emergency settings where time constraints are critical. The RTMDet marks a significant step forward in applying AI to enhance the speed and accuracy of medical diagnostics. Its development reflects broader trends in healthcare technology, where the goal is not only to achieve high accuracy but also to ensure that solutions are feasible and effective in real-world medical settings. As AI continues to evolve, it is expected that models like RTMDet will play an increasingly vital role in shaping the future of medical diagnostics.

The development of the RTMDet represents a significant step forward in the application of deep learning to medical diagnostics. By balancing computational efficiency with diagnostic accuracy, the RTMDet enhances the capabilities of medical imaging technologies, offering promising improvements for patient outcomes through faster and more precise brain tumor detection. This model sets a new standard for clinical applications, where the demand for rapid and accurate diagnostics is continuously rising.

## 6. Conclusions

The RTMDet has demonstrated a significant advancement in merging artificial intelligence with medical imaging diagnostics. This study showcased RTMDet’s capabilities to exceed existing deep learning models in terms of sensitivity, specificity, and computational efficiency, thereby establishing a new benchmark for real-time medical diagnostic tools. RTMDet’s superior diagnostic accuracy, with sensitivity and specificity nearing perfection, underscores its potential to reliably detect brain tumors. Its efficient use of computational resources, characterized by a reduced number of parameters and floating-point operations, enables its deployment in real-time settings where rapid decision-making is crucial. These attributes make RTMDet particularly relevant in clinical environments where timely and accurate diagnosis can significantly influence treatment outcomes. Despite these achievements, RTMDet’s journey is not complete. The model will benefit from further refinements to enhance its robustness across different clinical scenarios and imaging techniques. Increasing the interpretability of the RTMDet is also essential for its adoption in clinical settings, as practitioners value understanding how diagnostic conclusions are reached. Moreover, incorporating multimodal imaging data could broaden the diagnostic capabilities of the RTMDet, making it a more versatile tool in medical diagnostics. The RTMDet exemplifies the transformative potential of integrating advanced AI technologies in healthcare. It not only improves diagnostic processes but also has the potential to enhance patient management and care. The future development of the RTMDet will also involve adapting the model for various medical imaging modalities. This adaptation, achieved through transfer learning techniques, will extend the model’s utility to other crucial medical fields such as cardiology and orthopedics. Collaboration with radiologists and medical technologists will also be deepened to refine RTMDet, ensuring that it integrates seamlessly into existing diagnostic workflows. These collaborations are vital for incorporating practical insights and adapting the model to meet real-world needs.

## Figures and Tables

**Table 1:** Summarizing the key aspects of the RTMDet Model.

Aspect	Description
Foundation of CNNs	Built on CNNs, the RTMDet utilizes multiple convolutional layers to extract and interpret spatial hierarchies in image data, enabling effective object recognition at various scales and orientations.
Integration of Depthwise Convolutional Blocks	Incorporates depthwise separable convolutions (DWConvBlocks), where depthwise convolutions handle spatial feature extraction and pointwise convolutions adjust dimensionality. This reduces computational complexity while maintaining high feature resolution.
Real-Time Processing Capabilities	Optimized for real-time performance with computational techniques such as batch normalization and skip connections. These enhancements improve training efficiency and gradient flow, preventing vanishing gradient issues in deep networks.
Adaptations for Medical Imaging	Fine-tuned for detecting brain tumors by adjusting filter sizes and strategically placing depthwise blocks. These refinements enhance sensitivity to medical-specific features, improving detection precision.
Deployment and Practical Applications	Designed for integration into medical imaging systems, the RTMDet enables real-time analysis and alerts for early disease detection. Beyond medical applications, its adaptable architecture supports various real-time object detection tasks.

**Table 2:** The explanation by layers of the backbone part of the model.

$Layer_{1}$	$Layer_{2}$	$Layer_{3}$	$Layer_{4}$	$Layer_{5}$
$F_{CM1}\left(x_{input}\right)$ ↓ $F_{CM2}\left(F_{CM1}\right)$ ↓ $F_{CM3}\left(F_{CM2}\right)$ ↓ $F_{CM4}\left(F_{CM3}\right)$ ↓ $F_{CM5}\left(F_{CM4}\right)$	$F_{CM6}\left(F_{CM5}\right)$ ↓ $F_{CSPL1}\left(F_{CM6}\right)$	$F_{CM7}\left(F_{CSPL1}\right)$ ↓ $F_{CSPL2}\left(F_{CM7}\right)$	$F_{CM8}\left(F_{CSPL2}\right)$ ↓ $F_{CSPL3}\left(F_{CM8}\right)$	$F_{CM9}\left(F_{CSPL3}\right)$ ↓ $F_{Bottleneck}\left(F_{CM9}\right)$ ↓ $F_{CSPL4}\left(F_{Bottleneck}\right)$

**Table 3:** Summary of experiment and analyses.

Aspect	Details
Datasets Used	BraTS, ANDI (Brain Tumor Segmentation and Artificial and Natural Dataset for Intracranial Hemorrhage)
Preprocessing Steps	Skull stripping, image normalization, resampling, augmentation
Model Tested	RTMDet
Comparison Models	YOLOv5, YOLOv6, YOLOv7, YOLOv8, etc.
Key Metrics Evaluated	Average precision (AP), sensitivity, specificity, computational efficiency (FLOPs, parameters)
Main Outputs	RTMDet showed superior performance in speed and accuracy, with detailed results highlighted in the sensitivity and AP metrics.
Software and Tools	Description of software and analytical tools used for data processing and analysis.

**Table 4:** Summarizing the preprocessing steps in brain tumor detection using the BraTS and ANDI datasets.

Preprocessing Step	Description
Image Normalization	Ensures uniform intensity scales across different imaging modalities. MRI scans undergo Z-score normalization, adjusting pixel values to have a mean of zero and a standard deviation of one. CT scans use windowing to highlight intensity ranges relevant to hemorrhages.
Resampling	Standardizes resolution across all images to ensure consistent voxel sizes. This step enables the model to learn scale-invariant features and prevents variations in voxel dimensions from affecting training.
Skull Stripping	Removes non-brain tissues, particularly in MRI scans, to focus the model’s attention on brain structures where tumors are located. Automated algorithms leveraging anatomical atlases or deep learning methods are used for precision.
Augmentation	Enhances model robustness and prevents overfitting by applying rotation, flipping, and slight translations. These transformations help the model generalize better to variations present in clinical settings.

**Table 5:** The comparison results of the SOTA detection models for brain tumor detection.

Model	Dataset	Input Shape	Params (M)	FLOPs (G)	AP	Epochs
Yolov5	BraTS + ANDI	240 × 240	7.2	9.6	0.816	300
Yolov6	BraTS + ANDI	240 × 240	17.1	22.2	0.83	300
Yolov7	BraTS + ANDI	240 × 240	34.9	45.7	0.82	300
Yolov8	BraTS + ANDI	240 × 240	42.4	54.2	0.781	300
Yolov9	BraTS + ANDI	240 × 240	44.2	57.8	0.842	300
Yolov10	BraTS + ANDI	240 × 240	48.6	62.3	0.85	300
Yolov11	BraTS + ANDI	240 × 240	52.3	67.1	0.858	300
Baseline	BraTS + ANDI	240 × 240	8.99	14.8	0.846	300
RTMDet (Ours)	BraTS + ANDI	240 × 240	6.76	9.65	0.969	300

**Table 6:** Performance comparison of various models for brain tumor detection.

Model	Sensitivity	Specificity	AUC	Parameters (Millions)	FLOPs (Billions)
RTMDet (Ours)	0.95	0.94	0.95	6.5	9.1
Modified RetinaNet [5]	0.90	0.88	0.90	61.5	65.9
Attention-Fused MobileNet-LSTM [12]	0.93	0.90	0.93	44.0	38.6
Transfer learning-based [16]	0.92	0.91	0.92	7.3	9.8
Modified InceptionV3 [17]	0.87	0.85	0.88	25.6	15.2
MN-V2/CFO [21]	0.83	0.82	0.83	7.0	8.2
TumorAwareNet [22]	0.89	0.89	0.87	50.4	58.3
Modified MBConvFinetuned-B0 [23]	0.84	0.85	0.85	32.8	54.0
WSSOA [24]	0.88	0.85	0.88	21.9	35.4
DEF-SwinE2NET [25]	0.90	0.90	0.88	48.0	56.5
PyQDCNN [26]	0.91	0.90	0.90	29.6	39.1

## Data Availability

All of the used datasets are available online.
